# Exploring non-linear transition pathways in social-ecological systems

**DOI:** 10.1038/s41598-020-59713-w

**Published:** 2020-03-05

**Authors:** Jean-Denis Mathias, John M. Anderies, Jacopo Baggio, Jennifer Hodbod, Sylvie Huet, Marco A. Janssen, Manjana Milkoreit, Michael Schoon

**Affiliations:** 1 Université Clermont Auvergne, INRAE, UR LISC, F-63178 Aubière, France; 2 School of Sustainability, Arizona State University, Wrigley Hall, 800 Cady Mall 108, Tempe, AZ 85281 United States of America; 3 School of Human Evolution and Social Change, Arizona State University, Tempe, AZ 85281 United States; 4 Center for Behavior, Institutions and the Environment, Arizona State University, Tempe, AZ 85281 United States; 5 School of Politics, Security and International Affairs, University of Central Florida, Orlando, 32816 United States; 6 Sustainable Coastal System Cluster, National Center for Integrated Coastal Research, University of Central Florida, Orlando, 32816 United States; 7 Department of Community Sustainability, Michigan State University, 480 Wilson Road Room 310 B, East Lansing, MI 48824 United States of America; 8 Department of Political Science, Purdue University, 100 N University Street, West Lafayette, IN 47906 United States of America

**Keywords:** Psychology and behaviour, Sustainability

## Abstract

Tipping point dynamics are fundamental drivers for sustainable transition pathways of social-ecological systems (SES). Current research predominantly analyzes how crossing tipping points causes regime shifts, however, the analysis of potential transition pathways from these social and ecological tipping points is often overlooked. In this paper, we analyze transition pathways and the potential outcomes that these may lead to via a stylized model of a system composed of interacting agents exploiting resources and, by extension, the overall ecosystem. Interactions between the social and the ecological system are based on a perception-exploitation framework. We show that the presence of tipping points in SES may yield counter-intuitive social-ecological transition pathways. For example, the high perception of an alarming ecological state among agents can provide short-term ecological benefits, but can be less effective in the long term, compared to a low-perception condition. This work also highlights how understanding non-linear interactions is critical for defining suitable transition pathways of any SES.

## Introduction

Human actions and ecological systems are continually co-evolving via complex, interdependent feedback dynamics, making it difficult to fully understand such interactions between the social system and the ecological system, especially if each system is studied in isolation^1–5^. In addition, the non-linear processes that characterize both social and ecological dynamics reduce the ability of researchers and decision-makers to identify management strategies that maintain or restore the sustainability of social-ecological systems (SES)^6^. Although several studies^7–9^ analyze these non-linear processes, identifying transition pathways of SES from one state to another presents a major current challenge for managing SES. Identifying the set of available transition pathways is vital for assessing the impact of perturbations on SES sustainability as well as developing restoration policies for overexploited ecosystems. While tipping points have been widely studied^6,10–12^, there is a need to better assess their effects on potential and specific transition pathways of SES.

Tipping-point dynamics are generally analyzed as drivers of regime shifts, especially from an ecological point of view^6,11^. The definition of a tipping point slightly differs from one discipline to another. In physics, a tipping point is defined as an unstable equilibrium, whereas the concept is more qualitative in other disciplines. Generally a tipping point is characterized by the fact that “a small perturbation can cause a qualitative change in the future state of a system”^10^. Taking the broader literature into account, Milkoreit and colleagues^12^ enumerated over 20 definitions of tipping points. In this work we use the tipping point definition proposed by Milkoreit and colleagues^12^: a tipping point is “*the point or threshold at which small quantitative changes in the system trigger a non-linear change process that is driven by system-internal feedback mechanisms and inevitably leads to a qualitatively different state of the system, which is often irreversible*”^12^.

The study of transition pathways in SES through the lens of Ecological Tipping Points (ETP) and Social Tipping Points (STP) has received less attention because it requires formal SES modeling approaches that can account for social and ecological tipping processes. This is mainly due to the fact that (1) integrating social behaviour in ecological modeling is still a major challenge and (2) STP have been under-analyzed in terms of formal models. Integration of social behaviour in models has been a prominent question since the 1970’s^13^ with several works since in environmental sciences^1,14–17^. Whereas this is an “old” issue in some ways, it is quite new for analyzing regime shifts and tipping points in SES. Indeed, despite extensive efforts to develop coupled SES models, the majority of “regime shift” models focuses only on one linkage, either E → S or S → E, such as the work of Horan *et al*.^18^ on the influence of institutional changes on ecological regime shifts, the influence of individual profits on critical transitions^19^, or the analysis of early warnings in human-environment systems^20^.

**Figure 1:**
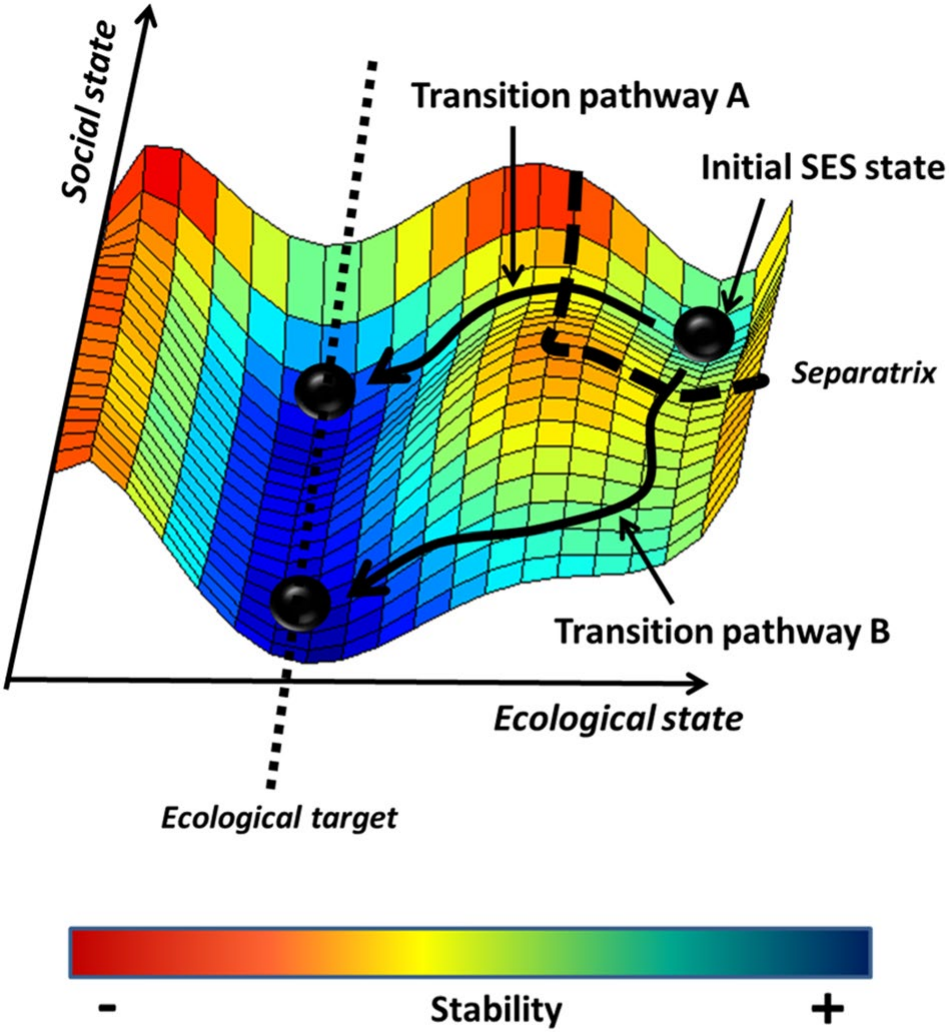
Social-ecological feedbacks for defining transition pathways in SES. In order to reach a given target state, several transition pathways are possible. Here, an ecologically driven transition pathway A and a socially driven transition pathway B are represented.

For analyzing how tipping points may affect transition pathways of SES, simple models of stability landscapes (as the one represented in Fig. 1) may provide important insights. For instance, consider an initial SES state and an ecologically motivated target SES state (see Fig. 1). The existence of a separatrix (a set of possible social-ecological tipping points) makes it difficult to identify how the target state can be reached. We explore how the available transition pathways can be represented in such a stability landscape. The first type of transition is represented by pathway A: an ecological source of change pushes the system over an ecological tipping point; there is no change in the social dimension of the system. However, the initial source of change may be social as represented by pathway B. In this case, a social and ecological tipping point is being crossed, transforming the SES. The target state can be reached along two different pathways, potentially open to different decision-making strategies. Other scenarios could involve a single STP or ETP opening up multiple pathways to end states that differ both in their social and ecological qualities.

Thus, in order to analyze more deeply how STP and ETP may influence non-linear transitions of SES, we created a formal model that takes both social and ecological processes and their feedbacks into account and identifies their effect on the selection of a pathway.

## A stylized social-ecological model of exploitation

The purpose of this stylized model is to highlight the effect of social-ecological feedbacks - how the dynamics of the ecological system affects the dynamics of social system and *vice versa* - and tipping points on the transition pathways of SES. More specifically, both the social and the ecological submodels of a coupled SES should have the potential for tipping behavior if we want to analyze feedbacks and spill-overs between them. Hence we devise a model with three key properties: tipping points characterize (1) the ecological submodel as well as the (2) social submodel and (3) where the social and the ecological submodels exhibit a double linkage in terms of dynamics. For the first two properties, we chose well-known models that exhibit ETP and STP. More specifically, we used a bioeconomic model^21–23^ for ETP that has been used for connecting institutional change and ecological tipping points^18^. Among social models, many exhibit STP, including Schelling’s spatial segregation model^24^, the critical mass model^25^ or opinion dynamics^26,27^. We opted for an opinion dynamic model based on a bounded confidence^26,28^ because of its simplicity (one equation, see Methods) and the presence of STP^28,29^. We created two-directional model coupling (i.e. E → S and S → E) through a perception-exploitation framework, which enables an analysis of unexpected social-ecological feedbacks within the SES (see Methods for a full description of the model). The model presented here focuses on interactions between two well-studied sub-models. The interactions between the ETP and STP models highlight the complexity of emerging transition pathways. Understanding this simple model is key in order to then assess more complex models that may include markets or institutional arrangements.

**Figure 2:**
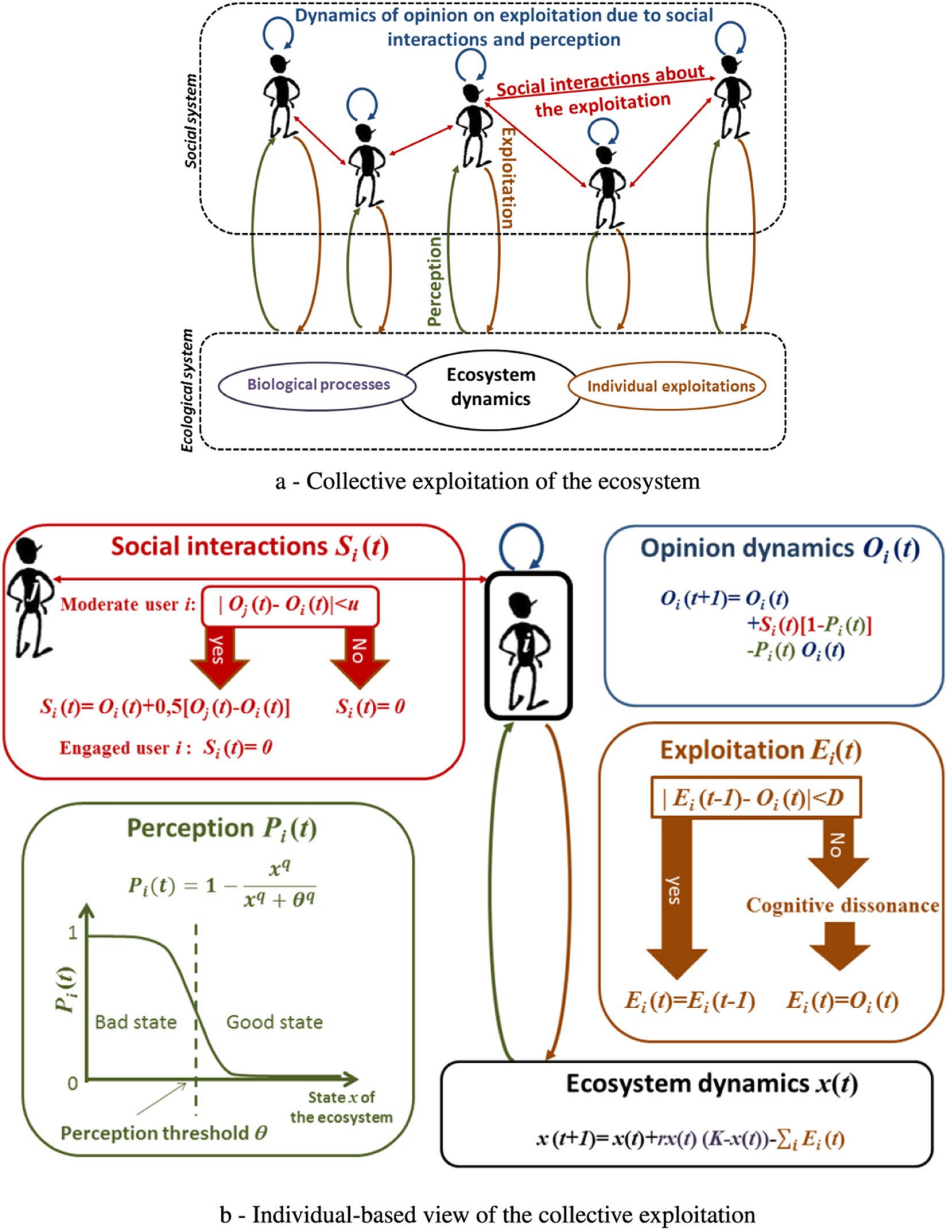
Schematic view of social-ecological interactions based on the collective (**a**) and individual (**b**) points of view. The global exploitation of the ecological system depends on the sum of individual exploitations (**a**) that depends on several social processes (perception, social interactions, etc.) that yields changes in individual options (**b**).

In a typical common-pool resource management context (see Fig. 2), resources are rivalrous (i.e. if one individual exploits the resource, the resources available to other users diminishes) but non-excludable (i.e. it is not possible to exclude individuals belonging to a community from exploiting the resource as rights are not individually owned but are defined at the community level). Here, we consider a community where no restrictions on resource exploitation exist. However, individuals can voluntarily reduce or increase resource exploitation depending on whether they perceive that the ecosystem is in an alarming state (i.e. is close to collapsing) or not (i.e. is in good condition). The interaction between exploitation and perception of ecosystem conditions is a key feature of our model. More precisely, we assume that several users exploit an ecosystem (orange arrow on Fig. 2). They tune their exploitation decisions according to their own opinion concerning the “right” level of resource harvest (blue arrow on Fig. 2). This means that individuals will exploit the system based on their own beliefs regarding the maximum possible harvest (i.e., individual benefit) that maintains a certain level of biomass, which enables them to maintain exploitation in the future.

If the results of these exploitation choices do not conform with their exploitation opinion, users will adapt and change their behavior in order to more closely match their opinion and actual exploitation results^30^. Further, aside from individual exploitation decisions, the dynamics of users’ opinions depend on a range of social processes based on specific individual characteristics, the overall social system and the ecological system (see also^31^). Here - for the sake of simplicity - we will not consider the full complexity of individual decision processes, but instead we focus on: (1) their interactions with the ecological system based on the perceived health of the ecosystem; (2) their interactions with the social system based on the opinions of other users about the right level of individual exploitation of the ecosystem; (3) individuals’ characteristics based on their opinion about the exploitation of the ecosystem. The health of the ecosystem is assessed by users by way of their perceptions of whether the ecosystem is in an alarming state (green arrow on Fig. 2). This perception process may aggregate a diversity of social processes based on the availability of information or on the knowledge infrastructure^32^, the social environment^33^, the cultural values of the population as well as the personal beliefs about undesirable ecological states. This perception process is tuned by a threshold *θ* above which an agent becomes alarmed about the state of the ecosystem (“high perception state”, see green box on Fig. 2b as well as Methods). All these social processes involved in perception are aggregated here as black box that amplify or reduce the state of being alarmed about the ecosystem. Concretely, a lower biomass level of the ecosystem is required for alarming agents with low perception.

Building on existing research on behavioral dynamics and collective action^34,35^, the social processes considered here concern interactions among users about their opinion on the right individual exploitation level (red network on Fig. 2). For representing such social interactions, we use the bounded confidence model^26^ (see red box on Fig. 2b as well as Methods).

Based on the perception of alarming ecological states and interaction with other users, users’ opinions evolve over time leading to changes in their exploitation levels (blue box on Fig. 2b, see also Methods). Finally, users change their exploitation behavior if their opinions are too far away from their current exploitation in order to avoid cognitive dissonance (see orange box on Fig. 2b and Methods).

This simple framework has the merit of being a generic approach to decision-making based on general feedbacks between social and ecological processes unfolding in a SES. Note that we have set up all parameters of the models within the range of parameters exhibiting STP and ETP. Values were extracted from the literature^21–23,29^ (see Methods for more details). We have chosen the values arbitrarily within these ranges but other values do not change the qualitative conclusions of the present work - as long as the values are within these tipping ranges.

Finally, we consider three types of users having different social-economic behavior in the system: moderate users, productive users and ecological users. Each user type is characterized by different (a) perception of alarming ecological states, (b) initial levels of ecosystem exploitation preferences, and (c) susceptibility to changes in opinion based on social interactions. Moderate users exhibit moderate behavior in terms of perception of alarming ecological states and exploitation. Productive users are characterized by a low perception - it means that they don’t decrease their exploitation even if the biomass is low - and a high initial level of exploitation. Ecological users are characterized by a high perception of - it means that they take into account the level of biomass in their decision process if the biomass is too low - and a low initial exploitation level. Further, while moderate users modify their opinions on the ecological state and exploitation via social interactions, productive and ecological users do not. That is, productive and ecological users have "extreme” opinions not susceptible to change via social interactions. Note that, in what follows, a part of the analysis is done by comparing the behavior of the moderate users with the behavior of the engaged users (productive and ecological ones), the latter being not sensitive to social interactions with two different perceptions of the ecosystem. Table 1 summarizes users characteristics. Each user has a social-economic behavior which is the same all along a simulation.

**Table 1:** User characteristics related to the suggestibility to change opinions, characteristics of the perception of the ecosystem health and initial level of exploitation.

Characteristics	Moderate users	Productive users (Engaged)	Ecological users (Engaged)
Susceptibility of users to change opinions by social interactions	Yes	No	No
**Perception of alarming ecological states**	Moderate	Low	High
Initial exploitation and opinion about exploitation	Moderate	High	Low

The purpose is to see how this diversity of social-economic behaviors may lead to sudden changes within the social-ecological systems and to analyze the involved transition pathways. For this purpose, we especially analyze two main issues with an increasing complexity: (1) how social changes may drive unexpected ecological transition pathways and (2) how ecological changes may drive coupled social and ecological transition pathways.

## Results

### Social tipping points as a driver of ecological transition pathways

We first focus on transition pathways with exclusively social drivers of change (as the transition pathway B on Fig. 1). We suppose that there is no perception of alarming ecological states and that opinions of users only change according to social interactions. In practice, no perception may mean either: (1) users do not have any information about the state of the ecosystem because of a lack of information infrastructure or a lack of environmental incentive (but they may be active if they would have the information); or (2) users have the information but they never perceive the ecosystem as within an alarming state because of their beliefs about undesirable ecological states. Thus, the three types of users defined in Table 1 still exist but they are not defined through the "perception of alarming ecological states" dimension. Indeed, without a perception process, opinion dynamics of the population only depend on social interactions. As explored in the literature, many final states may emerge, such as (a) moderate cluster(s), a single extreme, double extremes or continuously fluctuating opinions^29^.

With the values of parameters chosen here, previous work^28,29^ has shown that the presence of such engaged users may create instability and generate tipping processes, even if the proportion of engaged users is very low. Specifically, a new stationary state of this model has been highlighted that is particularly relevant to our topic, in which uncertain agents avoid converging to extreme positions: depending on uncertainty and/or the proportion of extremists, their opinions keep fluctuating instead of becoming extreme^28^. Besides, in some cases, we may observe a tipping behavior of the population: before converging to a single extreme, the population first converges to a quasi-stationary state of fluctuating opinions^28^.

**Figure 3:**
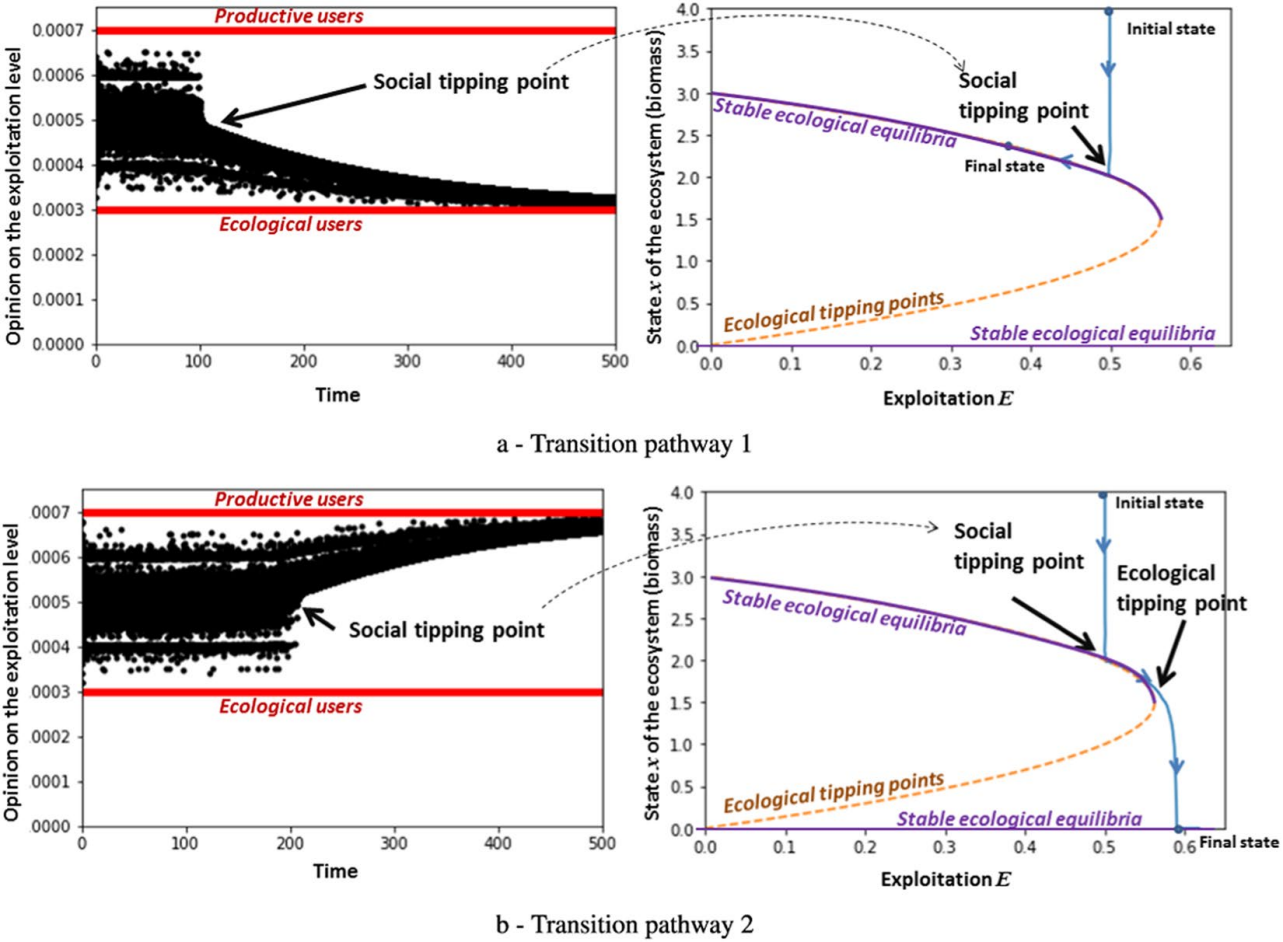
Transition pathways based on social dynamics that yield two final ecological states. In both cases, there is a sudden social change of the state (around *time* = 120 for the first transition pathway and *time* = 200 for the second one) due to instability of the system (left figures): opinions of the moderate users (in black) are fluctuating until a tipping towards engaged users (in red) depending on the tipping case: ecological users for case 1 (**a**) when ecological users have influenced all the moderate users and defined a strongly ecological norm, and productive users for case 2 (**b**) when productive users have influenced all the moderate users and defined a strongly productive behavior. According to the social tipping (productive or ecological tipping), we have two transition pathways: either there is a convergence towards a stable biomass equilibrium (case 1, **a**) or an extinction of the biomass (case 2, **b**).

In what follows, engaged users represent 0.2% of the population and are equally distributed as follows: 0.1% of ecological users (with a low level of individual exploitation) and 0.1% of productive users (with a high level of individual exploitation). In Fig. 3a,b, we have represented two transition pathways of our SES that depend on social tipping. In the left panel, the opinions of the population over time are represented: black points represent opinions of the moderate users and red points represent the opinions of engaged users (ecological and productive users). Opinions of the the population keep fluctuating until a tipping point is reached. This social tipping leads to a polarization of the population towards two extreme cases (see the left figures in Fig. 3). In both cases, there is a sudden social change of the state (around time = 100 for transition pathway 1 and time = 200 for transition pathway 2) due to instability of the system: opinions of the moderate users (in black) are fluctuating until a tipping towards engaged users (in red) occurs depending on the tipping case: ecological users for the first transition pathway (Fig. 3a) and productive users for the second one (Fig. 3b).

This social tipping (of perceptions) changes the exploitation behavior of users and therefore the ecological dynamics (see Fig. S1 in SI). The ecological system may also exhibit tipping points - as is the case here - that may change the final stable equilibrium of the SES. When the social tipping is toward ecologically-inclined users, the system converges towards a stable biomass equilibrium (transition pathway 1, Fig. 3a - right part). On the other hand, when the social tipping is toward productive users, the system reaches an ecological tipping point yielding an extinction of the biomass produced by the ecological system and exploited by the users (transition pathway 2, Fig. 3b - right part).

To summarize, the type of social tipping point will determine whether the SES crosses an ecological tipping point or not. This simple example shows how it is necessary to explore transition pathways beyond the presence of tipping points. Here, the social-ecological interactions will lead to two very different ecological transition pathways although the tipping point was initially social.

### Perception of ecological changes as a driver of social and ecological transition pathways

**Figure 4:**
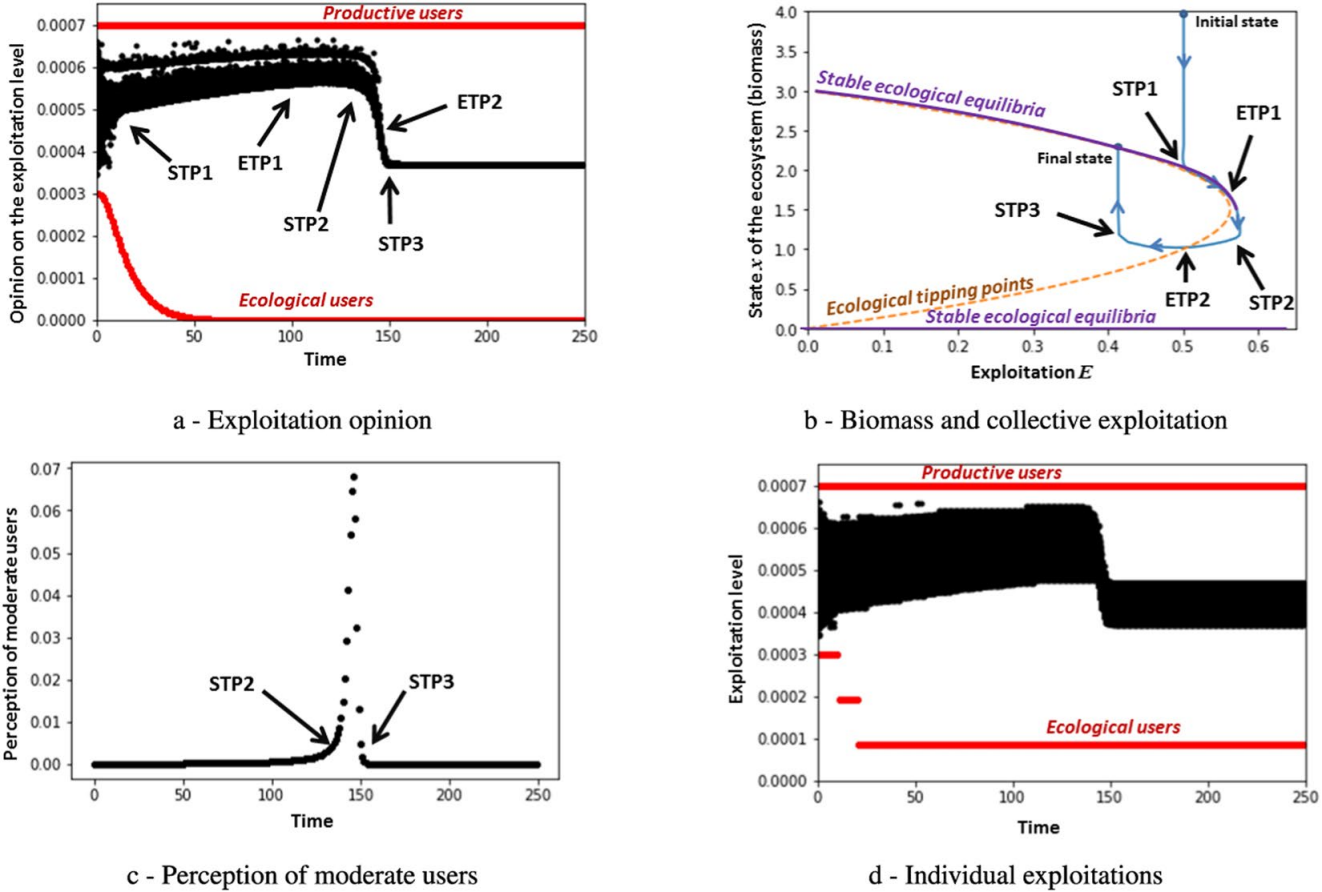
Socio-ecological transition pathway caused by socio-ecological feedbacks, social tipping points (STP) and ecological tipping points (ETP). For (**a,d**), red points represent engaged users whereas black points represent moderate users.

Now, we consider that users have a perception of alarming ecological states and that opinions of users change according to (1) these ecosystem state perceptions and (2) their social interactions. Due to this interaction between individual perceptions and social communication, user opinions can change in surprising ways. For example, although the exploitation opinions of moderate users are initially located between the opinions of ecological and productive users, their final exploitation opinion can be outside of this range, reaching the extreme lower limit (no exploitation). This is due to opinion adjustment dynamics described in the methods section. In this case, social tipping points are either due to social interactions (as it was the case in the previous section); or the perception of alarming ecological states. If we decrease the perception of all users, results are quite similar to the case described in the previous section (not represented here for the sake of simplicity). On the other hand, if we increase perception of all users, population tends to behave like ecological users. Note that the specific case of similar perception for all agents is described in SI “case of similar perception” (see the description as well as Fig. S10). Perception and exploitation of the ecosystem create feedbacks between the social and the ecological systems. Such feedbacks are portrayed in Fig. 4. The transition pathway is described as follows from a tipping point analysis:

- Initially, the high perception of alarming ecological states by ecological users leads them to decrease their individual exploitation to a low value (see Fig. S2 and the links between opinion and exploitation). This quick change of the opinion of ecological users creates a significant distance between ecological and moderate users, which implies that moderate users no longer adjust their opinions towards the ecological perspective. These dynamics yield an early social tipping point (STP1) of the opinions of moderate users towards the productive users;
- Corresponding increase of exploitation leads to a slow decrease of the biomass of the ecosystem (Fig. 4b) until the first ecological tipping point (ETP1). Biomass decrease even accelerates after ETP1;
- When the biomass reaches a low value (around 1), perception of moderate users change to high - they become alarmed about the state of the ecosystem. This leads to the second social tipping point (STP2, which is a perception tipping point (Fig. 4c)) with a significant decrease of the exploitation opinion (Fig. 4b) and actual level of individual exploitation because of the cognitive dissonance between users’ opinion and users’ exploitation results. This cognitive dissonance leads to delays as well as abruptness in the exploitation dynamics (see Fig. S3 for the influence of the cognitive dissonance on the results). Note that the level of biomass is not low enough to lead to a change of the opinion of the productive users;
- This decrease of exploitation, again created by changes among moderate users, leads the ecosystem to cross an ecological tipping point (ETP2) (Fig. 4b), after which the biomass of the ecosystem begins to increase;
- This increase of biomass changes the perception of moderate users again: they no longer perceive an alarming ecological state (Fig. 4c). This leads to the stabilization of their opinion (STP3, which is a perception tipping point) and of their exploitation levels (Fig. 4d). Note that this stabilization of the population is also due to the fact that moderate users are not influenced by ecological or productive users;
- Finally, this stabilization of opinions (and therefore of exploitations, see Fig. S2 in SI) leads to a convergence of the biomass (see the final state on Fig. 4b).

However, the inter-dependencies between the perception process and the ecological dynamics may yield counter-intuitive transition pathways in certain cases. For instance, we now increase or decrease the perception level of the whole population (engaged and moderate users) by adjusting which states of the ecosystem, i.e., levels of produced biomass, are considered alarming or secure. In other words, ecosystem states that were previously considered alarming are no longer alarming in new simulations (called Low perception in what follows compared to the reference Moderate perception represented in Fig. 4), or on the contrary, ecosystem states that were previously considered secure are perceived as alarming in new simulations (called High perception in what follows).

**Figure 5:**
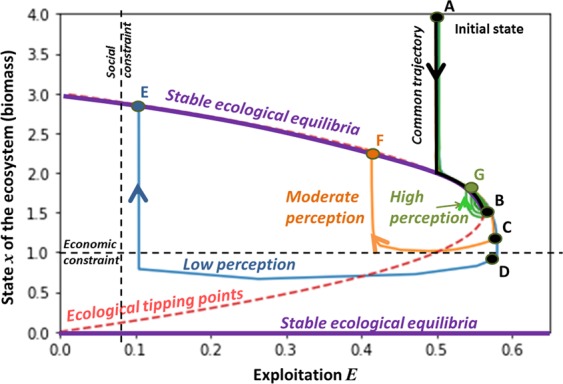
Influence of perception of alarming ecological states on ecological transition pathways. Despite of initial common trajectory (from point A to point B), perception level dramatically changes the trajectory of the ecological system. Three types of perceptions are tested. Low perception surprisingly leads to a higher final biomass (point E) whereas a high perception leads to a lower final biomass (point G). However, the minimum value of the biomass along the trajectory is lower for low perception compared to the trajectory induced by high perception.

Results are compared with the reference case (plotted in Fig. 4) in Fig. 5. Lower and higher perception of alarming ecological states follows the reference trajectory from the initial state (point A) to point B. Indeed, at point B, higher perception leads people to react sooner, i.e., to change their exploitation opinion, whereas lower perception leads people to wait for a lower biomass before reacting (points C and D). Surprisingly, delaying the reaction leads to higher final biomass (point E) than the final biomass of the moderate (point F) and high (point G) perception scenarios. This is due to the fact the SES crosses the ecological tipping points later, which enables the SES to reach a higher stable ecological equilibrium. Note that, in some cases, very low perception may cause the shutdown of the exploitation as is the case when we have no perception (see Fig. 3). Besides, the socio-ecological transition may exhibit complex behavior as it is the case for high perception: in this latter case, the system exhibits cyclic behavior before converging to point G. To explain this, let’s consider the opinion and perception dynamics (see also Fig. S7). Opinion of moderate users are attracted by productive users but their perception of the ecological state counter-balances this attraction yielding a stabilization of their opinion. Note that, if we continue simulations, there are two possible convergences (at infinite time) because of the perception process: either there is a long transient convergence (with a convergence of ${e}^{-1{0}^{-6}t}$, *t* being the time) or a rapid convergence towards a non-nil exploitation because of the cognitive dissonance that allows a non-nil exploitation (see Figs. S4, S5 and S6 in SI for more explanations).

Despite having a higher final biomass, low perception of alarming ecological states may momentarily push the SES towards undesirable states. For instance, let’s consider socio-economic constraints expressed by a minimum biomass (i.e. economic constraint on the Fig. 5) and a minimum exploitation (i.e. social constraint on the Fig. 5)^32,36^. These constraints are not implemented within the model and have been manually added on the figure to help with the following analysis. With a low perception of alarming ecological states, the economic constraint is not always complied with (see the blue trajectory on Fig. 5) whereas this economic constraint is complied with in the case of moderate and high perception. The moral of this simple example is that delaying critical state of the SES (because of weak perception) may yield an undesirable state but gives a favorable final state for the SES - a result perhaps different from what many expected! It shows the trade-offs between low and long term effects of transition pathways of SES: high perception produces short-term benefits with lower long-term benefits compared to low perception. Note that results have also been plotted in the biomass-opinion space (see Fig. S8).

### Implications for designing social-ecological policies in the Anthropocene

Defining sustainable transition pathways of SES still remains challenging in the context of global change because of highly non-linear interactions between the social and the ecological systems. Indeed, as discussed by Folke *et al*.^2^, SES intrinsically exhibit non-linear dynamics as well as potential tipping points, making the analysis of these transitions difficult. Despite of the importance of tipping points in transition pathways^37^, models have been prominently used for anticipating critical transitions^6^ and less used for exploring the set of potential transition pathways of SES in the presence of tipping points yielding unexpected non-linear feedbacks. Here, we focus on the non-linear interdependencies between the social and ecological entities in order to show how transition pathways emerge from non-linear feedbacks. Beyond coupling ecological and social models and analyzing STP and ETP, we especially focused on how socio-ecological interactions may influence the SES transitions in the short term as well as the long term. More specifically, results show that socio-ecological feedbacks may involve counter-intuitive ecological transition pathways because of the temporal scale of spillovers: a high perception of alarming ecological states provides short-term benefits but it is less effective in the long term, compared to low perception that may provide, on the other hand short-term negative externalities. For instance, in our example, our ecosystem is below the economic constraint threshold for an extended period (see Fig. 5) yielding negative externalities despite long term benefits. The low and high perception cases show the complexity of managing long-term and short-term benefits.

Taking into account such non-linear interactions is still challenging but necessary if we want to design suitable policy for managing SES as a whole. For instance, to cope with climate change, do we need first to invest in geoengineering technologies or to raise public awareness regarding the damages caused by climate change that may be a trigger for massive investment in geoengineering? More generally, exploring transition pathways highlights the role of norms, rules and values for managing SES^38,39^, which constitutes a key issue especially because “resource management is people management”^40^. Despite the importance of the social system for many global issues, social processes have been less theorized and modeled in the natural resource management literature as compared to the ecological components, limiting our understanding concerning social influences in SES transitions. However, even if understanding social dynamics remains challenging, beyond economic incentives, policies have to provide value-based reasons for people to change their expectations (and hence, behavior)^41^ and also to take into account social norms that play an important role for solving global issues such as climate change^42^. Finally, introducing more sophisticated economic and institutional processes, such as mediating behavior of markets or effort costs of exploitation may produce other perspectives in terms of transition pathways but the emerging complexity may make their analysis difficult. Therefore, both qualitative and quantitative tools are required in order to highlight social-ecological interactions, tipping points and hence transition pathways. Their integration will contribute to the design of social-suited policy, a key of success for solving large-scale contemporary issues.

## Methods

### General comments

The model has been implemented in python 3.6 with PyCuda. The code is available here: https://github.com/jdmathias/SES-transition-pathways.

We used a combination of existing models that exhibit tipping points for specific values of parameters. Therefore, we chose values of the parameters within this range for having tipping points. We used a discretized model with a unitary time step. The Fig. 2b represents a schematic view of the model.

### Ecosystem dynamics

The ecosystem dynamics, in our model, are represented by the classic model proposed by Clark^21^ discretized as follows:

1 $$x(t+1)=x(t)+B(x(t))-E(t)$$

Where the variable *x*(*t*) represents the biomass of the ecosystem at time *t* and depends on the level of exploitation *E*(*t*) and the ecosystem dynamics *B*(*x*(*t*)). Many studies^32,43–45^ have analyzed variations of the model system we represented in equation 1. Such analysis centered on robust management under different assumptions of ecosystem dynamics (*B*(*x*(*t*))) and ecosystem exploitation (*E*(*t*))^21,22^. In this model, we represent the biomass dynamics as follows:

2 $$B(x(t))=rx(t)(K-x(t))$$

where the parameter *r* represents the ecosystem regeneration capacity (set at *r* = 0.25), *K* represents the maximum carrying capacity (set at *K* = 3) of the ecosystem. The population is composed of *N* agents (*N* = 1000). Note that these parameters have been chosen in order to have ecological tipping points and therefore to avoid straightforward ecological dynamics. Note that others parameters values may be chosen as long as these values enable us to have ecological tipping points. The total ecosystem exploitation *E*, on the other hand, is given by the the sum of each individual user exploitation level as in^21– 23^ $E(t)={\sum }_{i=1}^{N}{E}_{i}(t)$. *N* is the total number of users (here we set *N* at 1000), and *E*_*i*_(*t*) is the exploitation level of user *i* at time *t*. *E*_*i*_(*t*) is then determined by one personal characteristics and its social interactions. Fig. 2 graphically shows the model system we employ here to analyze the interactions between social and ecological processes and their overall effect on the SES of interest. In what follows, the initial exploitation of productive users is set at 0.0007 whereas the exploitation of ecological users is set at 0.0003. Exploitations of moderate users are randomly set following ${\mathscr{N}}({m}_{E},{m}_{E}/10)$ with *m*_*e*_ = 0.0005. It yields an initial total exploitation *E*(0) = *N* × *m*_*E*_ = 0.5.

### From opinion to exploitation... and cognitive dissonance

In our stylized model, users form an opinion about the relevant behavior by perceiving the resource state, and discussing the right level of exploitation. They change their behavior when their opinion is perceived by them as significantly different, and their current behavior is thus assessed as irrelevant. In social psychology, it has been shown there is a strong correlation between someone’s behavior and someone’s opinion about this behavior^46^.

This change behavior decision is inspired from the Cognitive Dissonance theory of Festinger which argues that people suffer from a psychological discomfort^30^ when they perceive incoherence between their behavior and their beliefs, in our case the agents’ opinion and their behavior. Moreover, numerous authors have shown there is a lot of resistance to behavioral change in situations to which the “good” behavior is not already known by people. Messages can be neglected and/or distorted; the problem can be redefined, etc.^47–49^.

The opinion is computed accordingly to the theory of reasoned action^46^. We assume it depends on a subjective norm given by an “attitude” process based on discussions between agents, and personal beliefs corresponding to the personal experiment, in our model the already adopted behavior of the agent. Personal belief and subjective norm are weighted accordingly to oneself perception of the resource state: the more critical the resource state, the more weighted is the personal belief, the less weighted is the subjective norm. This means, in critical situations issued from a social process defining a behavior, agents do not trust anymore to society to define their behavior.

The “social interaction” process defining the subjective norm is built accordingly to the theory of the social comparison^50^ which has shown people ordinary refer to the social world to decide about an “unknown value”. In practice, they tend to adopt the others’ opinion until the point they feel close enough to them to perceive a quite consensual norm about this value. This is also in accordance with the theory of Social Judgement^51^ defining each agent has an “assimilation latitude” corresponding to a difference to others to which they feel close to them and assimilate them to us. This assimilation latitude is denoted *u* in our “social interaction” process.

Using a formal description, we consider that each user *i* has an exploitation opinion *O*_*i*_(*t*) about what she thinks to be the right exploitation according to the perceived state of the ecosystem and interactions with other users. In other words, this exploitation opinion *O*_*i*_(*t*) corresponds to a level of exploitation which results from her perception of the ecosystem and her interactions with other users. The exploitation *E*_*i*_(*t*) is connected to the exploitation opinion *O*_*i*_(*t*) as follows (see also the orange part on Fig. 2):

3 $${E}_{i}(t)=\left\{\begin{array}{ll}{E}_{i}(t-1), & \,{\rm{if}}\,\ | {O}_{i}(t)-{E}_{i}(t-1)| \ \le \ D\\ {O}_{i}(t), & \,{\rm{otherwise}}\,\end{array}\right.$$

where *D* corresponds to the dissonance threshold and is set to 0.0001. If the exploitation opinion *O*_*i*_(*t*) of user *i* corresponds to her current exploitation *E*_*i*_(*t* − 1) (i.e. *O*_*i*_(*t*) = *E*_*i*_(*t* − 1)), exploitation of user *i* is in harmony with her exploitation opinion. If her exploitation opinion *O*_*i*_(*t*) is far away from her exploitation *E*_*i*_(*t* − 1) (higher than *D*), there is a cognitive dissonance: user *i* doesn’t do what she thinks to be right and she may subjected to negative psychological consequences^30^. When there is an inconsistency between exploitation opinion and current exploitation (dissonance), something must change to eliminate this dissonance^30^: we consider that user *i* changes her exploitation *E*_*i*_(*t* − 1) when her exploitation is too far away from her exploitation opinion *O*_*i*_(*t*) (i.e., |*E*_*i*_(*t* − 1) − *O*_*i*_(*t*)| > *D*).

### Opinion dynamics

We simulate opinion dynamics by using the bounded confidence (BC) model^26,29^. The exploitation opinion *O*_*i*_(*t*) of user *i* changes according to (see the blue part on Fig. 2): 1) social interactions *S*_*i*_(*t*) with other users and 2) her perception process *P*_*i*_(*t*) of the state *x* of the exploited ecosystem yielding the following discretized dynamics (with a unitary time step):

4 $${O}_{i}(t+1)={O}_{i}(t)+\mathop{\underbrace{{S}_{i}(t)}}\limits_{{{\rm{Social}}\,{\rm{interactions}}}}-\mathop{\underbrace{{S}_{i}(t){P}_{i}(t)}}\limits_{{ \mbox{''} {\rm{Social}}\,{\rm{interactions/perception}}\mbox{''}\,{\rm{interaction}}}}-\mathop{\underbrace{{P}_{i}(t){O}_{i}(t)}}\limits_{{{\rm{Perception}}}}$$

- *S*_*i*_(*t*) are represented by BC model that consider bounded pair interactions: if user *i* interacts with user *j*, the user *i* is only influenced by user *j* if opinion user *j* is not too far away of her opinion - modeled by a threshold denoted *u*^26^. These social interactions are described more in detail in the next section;
- *P*_*i*_(*t*) represents the perception process of individuals about the alarming ecological state of the system and dynamically evolved according to the state *x* of the ecosystem. Perception process can be modelled as having an effect only when alarming. In other words, perception influences the social interactions and social dynamics if and only if there is a clear alarming perceived ecological environment.

According to Eq. 4, it is possible to weight the influence of social interactions via the perception function *P*_*i*_(*t*). This perception function evolves over time but is ranged between 0 and 1. Note that if the perception function takes the value of 0 (meaning no perception of alarming ecological state), the dynamics of user *i* will be only based on social interactions. On the other hand, if the perception function is equal to 1 (meaning that the perception of ecological state is very alarming): (1) social interactions will not influence the opinion of user *i*; (2) the exploitation opinion becomes 0, e.g. user *i* thinks that we need to stop exploitation of the ecosystem (limit case). Therefore, in whats follows, social tipping points aggregate: (1) tipping points caused by social interactions and (2) tipping points due to perception of the ecological state. Note that, despite users’ initial opinions are ranged between 0.0003 and 0.0007, but their opinions can evolve between 0 (nil exploitation) and 0.0007 (initial opinion of productive users).

### Social interactions: the bounded confidence model

For modeling social interactions *S*_*i*_(*t*), we use the bounded confidence model (see the red part on Fig. 2). Following the original model of^26^, we consider a population of *N* agents composed of two types of users:

- moderate users: they may be influenced by their interactions with other users (in opinion jargon, we have a high value of opinion’s uncertainty). All the moderate agents exhibit the same uncertainty *u* about their opinion: if a moderate user *i* discuss with a user *j* with an exploitation opinion *O*_*j*_(*t*) far away from her opinion (|*O*_*i*_(*t*) − *O*_*j*_(*t*)| > *u*), user *i* is not influenced by user *j*;
- engaged users: they are defined as people with an “extreme” opinion that never change due to social interactions.

Note that uncertainty *u* in opinion dynamics represents a “distance of opinion” threshold above which no interaction takes place. In other words, it represents an opinion range within which agent can be influenced: if an agent *i* (with opinion *O*_*i*_(*t*)) discussed with another agent *j* (with opinion *O*_*j*_(*t*)), there is an interaction only if |*O*_*i*_(*t*) − *O*_*j*_(*t*)| < *u*. The initial opinions of users are equal to their individual exploitations *E*_*i*_(*t*). Engaged user has a high certainty about its opinions (*u* = 0). The proportion of engaged users in the population is denoted *p*_*e*_ (*p*_*e*_ = 0.2% with 0.1% of ecological users and 0.1% of productive users). An engaged user exhibits the two following features: (i) it expresses an extreme opinion, either 0.0003 (low exploitation of ecological users) or 0.0007 (high exploitation of productive users); (ii) it has a high certainty about its opinions (it means that uncertainty of engaged users is null and that they do not change their opinion). Each user is paired once in one period: $\frac{N}{2}$ random pairs interact at each time step. When a user *i* (with opinion *O*_*i*_(*t*)) meets an agent *j* (with opinion *O*_*j*_(*t*)), opinion of user *i* is modified as follows:

5 $$\left\{\begin{array}{l}{S}_{i}(t)=0.5[{O}_{j}(t)-{O}_{i}(t)]\ {\rm{if}}\ {\rm{user}}\ i\ {\rm{is}}\ {\rm{moderate}}\ {\rm{AND}}\ {\rm{if}}\ | {O}_{i}(t)-{O}_{j}(t)|  < u\\ {S}_{i}(t)=0\ {\rm{if}}\ {\rm{user}}\ i\ {\rm{is}}\ {\rm{engaged}}\end{array}\right.$$

All individuals’ opinions are updated simultaneously at each time step yielding a simultaneous updating of the opinions. *u* is set at 0.00021 for moderate users and 0 for engaged users. The values of *u* and *p*_*e*_ have been chosen in order to have opinion tipping within a reasonable time range for computation (between time = 100 and 200)^29^: have shown that it depends on the combination of *u* and *p*_*e*_. Other combinations will not change qualitatively the conclusions of the paper as long as *u*/*p*_*e*_-combinations yield opinion tipping points.

### Perception

For the perception function *P*_*i*_(*t*), we choose a function based on a sigmoïd (s-shaped) which is equal to 0 in the case of no alarming perception of the ecological state and equal to 1 when the ecological state is catastrophic (see the green part on Fig. 2):

6 $${P}_{i}(t)=1-\frac{x{(t)}^{q}}{x{(t)}^{q}+{\theta }^{q}}$$

This function has been chosen because it enables to control the value of the perception inflection point (*θ*-parameter) as well as the rate of perception change (*q*-parameter set at 10). Indeed, the perception function *P*_*i*_(*t*) is controlled by the value of *θ* (set at 0.775) that represents the inflection point of the sigmoïd, i.e. it is the alarming state of the ecosystem for which the perception of moderate users suddenly increases in such a way they perceived the ecological state in an alarming state. Note that the exact value of the inflection point is $\theta {\left(\frac{q-1}{q+1}\right)}^{1/q}$ which is approximated by *θ*. Note that other functions may be used as long as it exhibits sudden change in perception. As explained before, the perception of ecological users is supposed to be higher (the *θ*-value of ecological users is multiplied by 2) whereas the perception of productive users is supposed to be lower (the *θ*-value of productive users is divided by 2). We set *q* = 10 that implies a high rate of perception change in order to have perception tipping. Note that other high values of *q* - yielding sudden perception change - will not change qualitatively the results. For Fig. 3, case of no perception, we fix the value of *θ* to 0: it means that the perception function does not evolve over time, i.e. *Pi*(*t*) = *cst* = 0. Figure 4 represents results of the reference scenario, i.e. *θ* = 0.9. For Fig. 5, we multiply the value of *θ* by 0.9 or 1.1 for testing the effect of perception leading to *θ* = 0.9*0.775 for "low perception” case and *θ* = 1.1*0.775 for the "high perception” case. The perception function is plotted on Fig. S9 for the three cases.

## Supplementary information

Supplementary Information.

Supplementary Information2.

## References

[1] Liu J (2007). Complexity of coupled human and natural systems. Science.

[2] Folke C (2002). Resilience and sustainable development: Building adaptive capacity in a world of transformations. Ambio.

[3] Turner BL (2003). A framework for vulnerability analysis in sustainability science. Proceedings of the National Academy of Sciences.

[4] An, L. Modeling human decisions in coupled human and natural systems: Review of agent-based models. *Ecological Modelling* **229**, 25–36 (2012). Modeling Human Decisions.

[5] Pahl-Wostl, C. *et al*. Social learning and water resources management. *Ecology and Society* **12** (2007).

[6] Scheffer M (2012). Anticipating critical transitions. Science.

[7] Filatova T, Polhill JG, van Ewijk S (2016). Regime shifts in coupled socioenvironmental systems: Review of modelling challenges and approaches. Environmental Modelling & Software.

[8] Scheffer M, Carpenter SR (2003). Catastrophic regime shifts in ecosystems: linking theory to observation. Trends in Ecology & Evolution.

[9] Folke C (2004). Regime shifts, resilience, and biodiversity in ecosystem management. Annual Review of Ecology, Evolution, and Systematics.

[10] Lenton TM, Williams HT (2013). On the origin of planetary-scale tipping points. Trends in Ecology & Evolution.

[11] Carpenter SR, Brock WA, Folke C, van Nes EH, Scheffer M (2015). Allowing variance may enlarge the safe operating space for exploited ecosystems. Proceedings of the National Academy of Sciences of the United States of America.

[12] Milkoreit M (2018). Defining tipping points for social-ecological systems scholarshipâan interdisciplinary literature review. Environmental Research Letters.

[13] Bossel H, Strobel M (1978). Experiments with an âintelligentâ world model. Futures.

[14] Janssen M, de Vries B (1998). The battle of perspectives: a multi-agent model with adaptive responses to climate change. Ecological Economics.

[15] Castilla-Rho JC (2017). Social tipping points in global groundwater management. Nature Human Behaviour.

[16] Jager W, Janssen M, Vries HD, Greef JD, Vlek C (2000). Behaviour in commons dilemmas: Homo economicus and homo psychologicus in an ecological-economic model. Ecological Economics.

[17] Schlüter M (2017). A framework for mapping and comparing behavioural theories in models of social-ecological systems. Ecological Economics.

[18] Horan RD, Fenichel EP, Drury KLS, Lodge DM (2011). Managing ecological thresholds in coupled environmental–human systems. Proceedings of the National Academy of Sciences.

[19] Richter A, Dakos V (2015). Profit fluctuations signal eroding resilience of natural resources. Ecological Economics.

[20] Bauch CT, Sigdel R, Pharaon J, Anand M (2016). Early warning signals of regime shifts in coupled human–environment systems. Proceedings of the National Academy of Sciences.

[21] Clark C (1973). The economics of overexploitation. Science.

[22] Clark C, Gordon R (1975). The economics of fishing and modern capital theory: a simplified approach. Journal of environmental economics and management.

[23] Walters, C. *Adaptive management of renewable resources*. (New-York: McGraw Hill., 1986).

[24] Schelling, T. *Micromotives and Macrobehavior* (UK: Cambridge Univ Press, 1978).

[25] Granovetter M (1978). Threshold models of collective behavior. American Journal of Sociology.

[26] Deffuant, G., Amblard, F., Weisbuch, G. & Faure, T. How can extremism prevail? a study based on the relative agreement interaction model. *Journal of Artificial Societies and Social Simulation* **5** (2002).

[27] Hegselmann R, Krause U (2002). Opinion dynamics and bounded confidence: models, analysis and simulation. Journal of Artificial Societies and Social Simulation.

[28] Mathias J-D, Huet S, Deffuant G (2017). An energy-like indicator to assess opinion resilience. Physica A: Statistical Mechanics and its Applications.

[29] Mathias J-D, Huet S, Deffuant G (2016). Bounded confidence model with fixed uncertainties and extremists: The opinions can keep fluctuating indefinitely. Journal of Artificial Societies and Social Simulation.

[30] Festinger, L. *A theory of cognitive dissonance* (Stanford, CA: Stanford University Press, 1957).

[31] Baggio JA, Hillis V (2018). Managing ecological disturbances: Learning and the structure of social-ecological networks. Environmental Modelling & Software.

[32] Anderies, J. M., Mathias, J.-D. & Janssen, M. A. Knowledge infrastructure and safe operating spaces in social–ecological systems. *Proceedings of the National Academy of Sciences* (2018). 10.1073/pnas.1802885115 PMC6431179 30111542

[33] Bousquet F (1993). Simulating the interaction between a society and a renewable resource. Journal of Biological Systems.

[34] Bowles S, Gintis H (2002). Social capital and community governance*. The Economic Journal.

[35] Castillo D, Saysel AK (2005). Simulation of common pool resource field experiments: a behavioral model of collective action. Ecological Economics.

[36] Mathias J-D, Anderies JM, Janssen M (2018). How does knowledge infrastructure mobilization influence the safe operating space of regulated exploited ecosystems?. Earth’s Future.

[37] Westley F (2011). Tipping toward sustainability: Emerging pathways of transformation. AMBIO: A Journal of the Human Environment.

[38] Jones, N. A., Shaw, S., Ross, H., Witt, K. & Pinner, B. The study of human values in understanding and managing social-ecological systems. *Ecology and Society* **21** (2016).

[39] Baggio J (2016). Explaining success and failure in the commons: the configural nature of ostrom’s institutional design principles. International Journal of the Commons.

[40] Berkes, F. & Folke, C. *Linking sociological and ecological systems: management practices and social mechanisms for building resilience*. (Cambridge University Press, New York, New York, USA, 1998).

[41] Young HP (2015). The evolution of social norms. Annual Review of Economics.

[42] Nyborg K (2016). Social norms as solutions. Science.

[43] Barbier EB, Strand I (1998). Valuing mangrove-fishery linkages - a case study of campeche, mexico. Environmental and Resource Economics.

[44] Costello C (2016). Global fishery prospects under contrasting management regimes. Proceedings of the National Academy of Sciences.

[45] Sanchirico JN, Wilen JE (2005). Optimal spatial management of renewable resources: matching policy scope to ecosystem scale. Journal of Environmental Economics and Management.

[46] Fishbein M (1979). A theory of reasoned action: Some applications and implications. Nebraska Symposium on Motivation.

[47] Gifford R (2011). The dragons of inaction: psychological barriers that limit climate change mitigation and adaptation. The American psychologist.

[48] Mintzberg H, Raisinghani D, Theoret A (1976). The structure of "unstructured" decision processes. Administrative Science Quarterly.

[49] Moser SC, Ekstrom JA (2010). A framework to diagnose barriers to climate change adaptation. Proceedings of the National Academy of Sciences.

[50] Festinger L (1954). A theory of social comparison processes. Human Relations.

[51] Sherif, M. & Hovland, C. *Social Judgment. Assimilation and contrast effects. Communication and attitude change* (New Haven and London: Yale University Press, 1961).

