# Supplementary material for: Exploring non-linear transition pathways in social-ecological systems
Source: Sci Rep. 2020 Mar 5;10:4136. doi: 10.1038/s41598-020-59713-w (PMC7058029; doi:10.1038/s41598-020-59713-w)
Supplement: Supplementary file 1 — Supplementary Information. [file 41598_2020_59713_MOESM1_ESM.pdf]

Beyond tipping points: exploring non-linear transition pathways of social-ecological systems: Supplementary Information: Figures S1, S2, S3, S4, S5, S6, S7, S8 and S9

Jean-Denis Mathias[1,*], John M. Anderies[2,3,4,+], Jacopo Baggio[5,6,+], Jennifer Hodbod[7,+], Sylvie Huet[1,+], Marco A. Janssen[2,4,+], Manjana Milkoreit[8,+], and Michael Schoon[2,4,+]

[1]Université Clermont Auvergne, Irstea, UR LISC, Centre de Clermont-Ferrand, F-63178 Aubière, France
[2]School of Sustainability, Arizona State University, Wrigley Hall, 800 Cady Mall 108, Tempe, AZ 85281, United States of America
[3]School of Human Evolution and Social Change, Arizona State University, Tempe, AZ 85281, United States
[4]Center for Behavior, Institutions and the Environment, Arizona State University, Tempe, AZ 85281, United States
[5]School of Politics, Security and International Affairs, University of Central Florida, Orlando, 32816, United States
[6]Sustainable Coastal System Cluster, National Center for Integrated Coastal Research, University of Central Florida, Orlando, 32816, United States
[7]Department of Community Sustainability, Michigan State University, 480 Wilson Road Room 310 B, East Lansing, MI 48824, United States of America
[8]Department of Political Science, Purdue University, 100 N University Street, West Lafayette, IN 47906, United States of America
[*]jean-denis.mathias@irstea.fr
[+]these authors contributed equally to this work

ABSTRACT

This Supplementary Information presents Figures S1, S2, S3, S4, S5, S6, S7, S8 and S9.

Social tipping points as a driver of ecological transition pathways

Figure S1 shows the dynamics of individual opinions and exploitations in the case of no perception (see also Figure 3 of the main text).

Perception of ecological changes as a driver of social and ecological transition pathways

Figure S2 shows the dynamics of individual opinions and exploitations in the case of distinct perceptions between moderate and engaged users (see also Figure 4 of the main text).

Influence of cognitive dissonance

Figure S3 shows the influence of the value of D (threshold for the cognitive dissonance) on the dynamics (see also Figure 5 of the main text).

Long-term simulations

Figures S4, S5 and S6 show long-term simulations of Figure 6. shows the influence of the value of D (threshold for the cognitive dissonance) on the dynamics (see also Figure 5 of the main text).

The system converges towards a stable equilibrium for two reasons: the final point corresponds to an ecological equilibrium (x-dimension) and an exploitation equilibrium (E-dimension). Ecological equilibria are well-known whereas the exploitation equilibrium depends on the opinion dynamics and the cognitive dissonance D (that links opinion and exploitation): if the exploitation is equal to a value lower than D, there is no cognitive dissonance and the opinion does not influence the exploitation anymore. Therefore, depending on when this low exploitation is reached, either we have a rapid convergence or we have a

long transient convergence. The rapid convergence towards an equilibrium (as point E plotted on Figure 5) is obtained when users have a low perception, leading to delay the decrease of their exploitation. This delay pushes users to reach a very low exploitation if they want to reach again an acceptable level of biomass. In this case (as point E on the Figure), the reached exploitation is lower than D, yielding a stable equilibrium. Now let's consider a moderate perception as the orange pathway represented on Figure 5. The point F is not a stable equilibrium as expected by reviewer because there is very long transient dynamics in this case. The driver of this long transient dynamics is due to the perception and the cognitive dissonance. Once the ecosystem has reached point F, the main driver of opinion dynamics is the perception process. The perception process in this case is very low leading to a decrease of opinion that follows $e^{-10^{-6}t}$ (this value can be found by replacing x by 2.5 in equation 6, 2.5 being the value of the biomass at point F). So the trajectory slightly follows the line of ecological equilibria.

Exploitation and perception in the case of high perception

Figure S7 shows the dynamics of individual opinions and perception of moderate users (see also Figure 5 of the main text).

Dynamics in the opinion-biomass space

Figure S8 shows the results of Figure 5 in the exploitation-biomass space and in the opinion-biomass space (see also Figure 6 of the main text).

Perception function

Figure S9 shows the perception function for low, moderate and high perceptions.

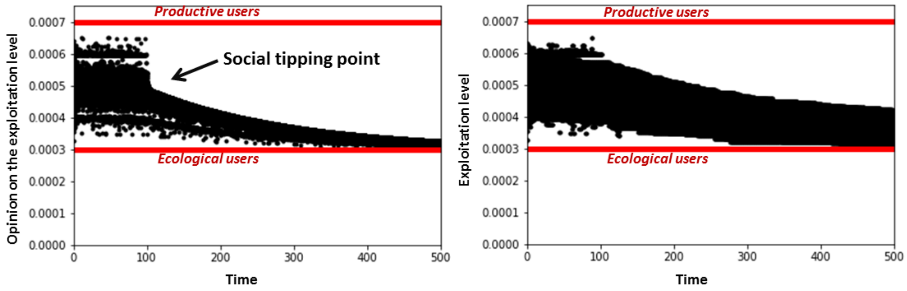

a - Without perception, case 1.

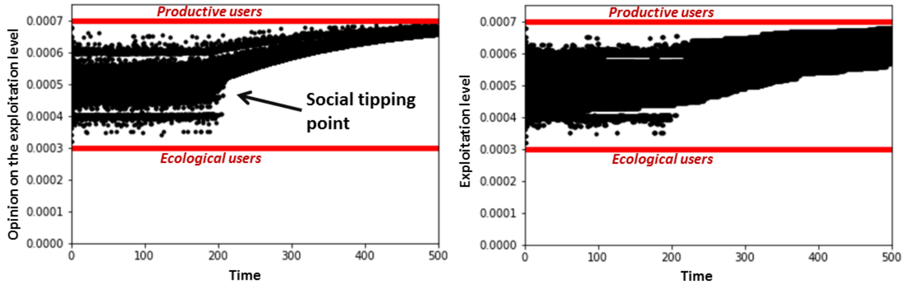

b - Without perception, case 2.

Figure S1: Opinion dynamics (left figures) and exploitation dynamics (right figures). Opinion dynamics changes the exploitation because of cognitive dissonance

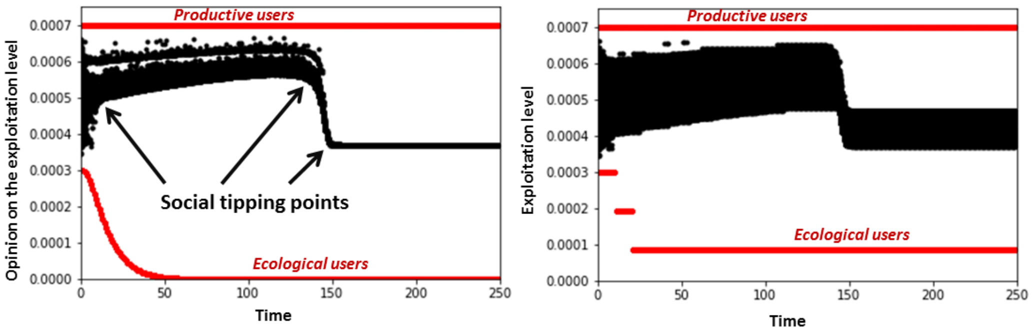

Figure S2: Opinion dynamics (left figure) and exploitation dynamics (right figure). Opinion dynamics changes the exploitation because of cognitive dissonance. Note that the opinions slightly decrease in the long term because of the (weak) negative perception of the ecological state

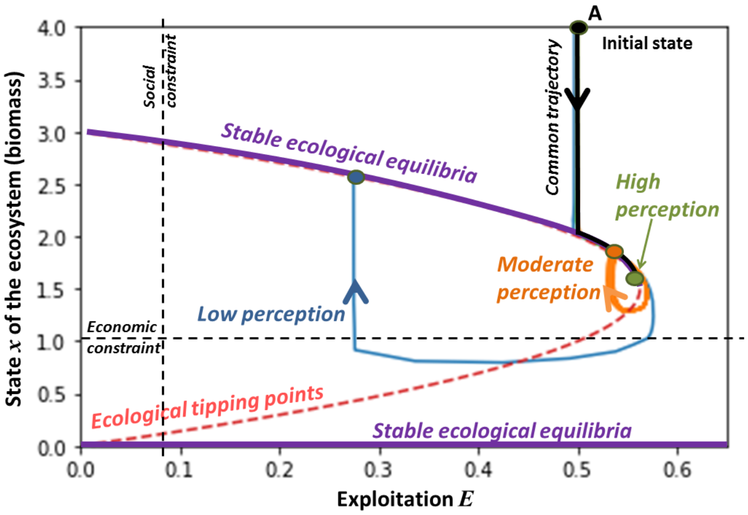

a - 25% of the initial value of D.

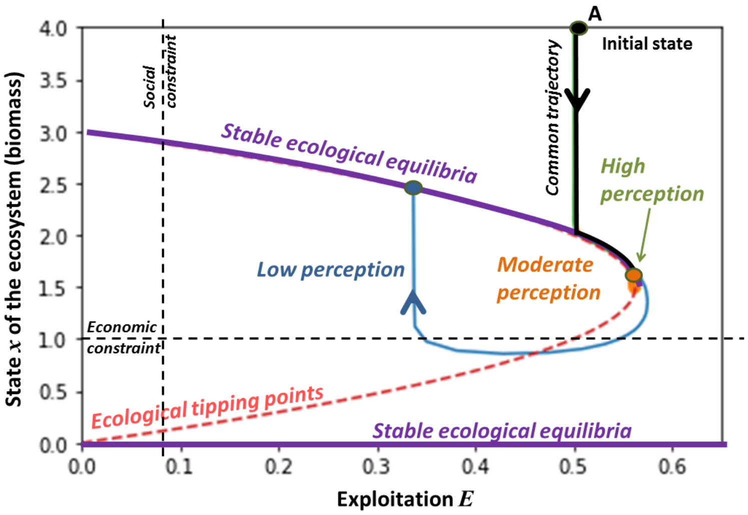

b - 5% of the initial value of D.

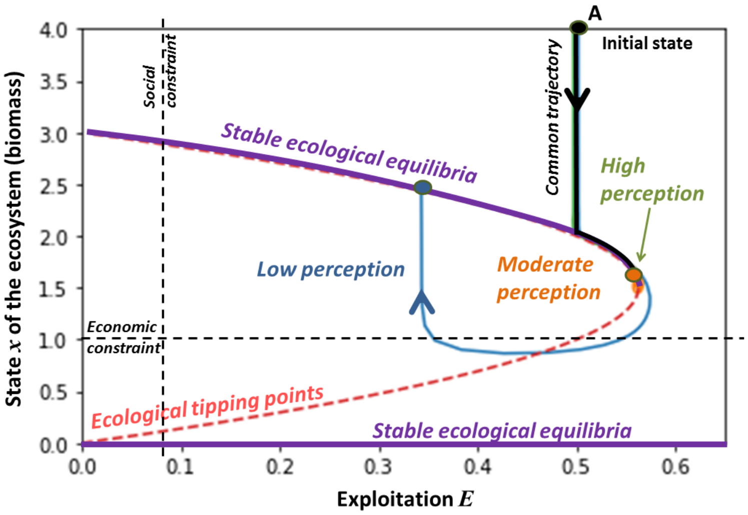

c - D=0, no cognitive dissonance.

Figure S3: influence of the value of D. Small values decrease the delayed effects and tend to smooth the results.

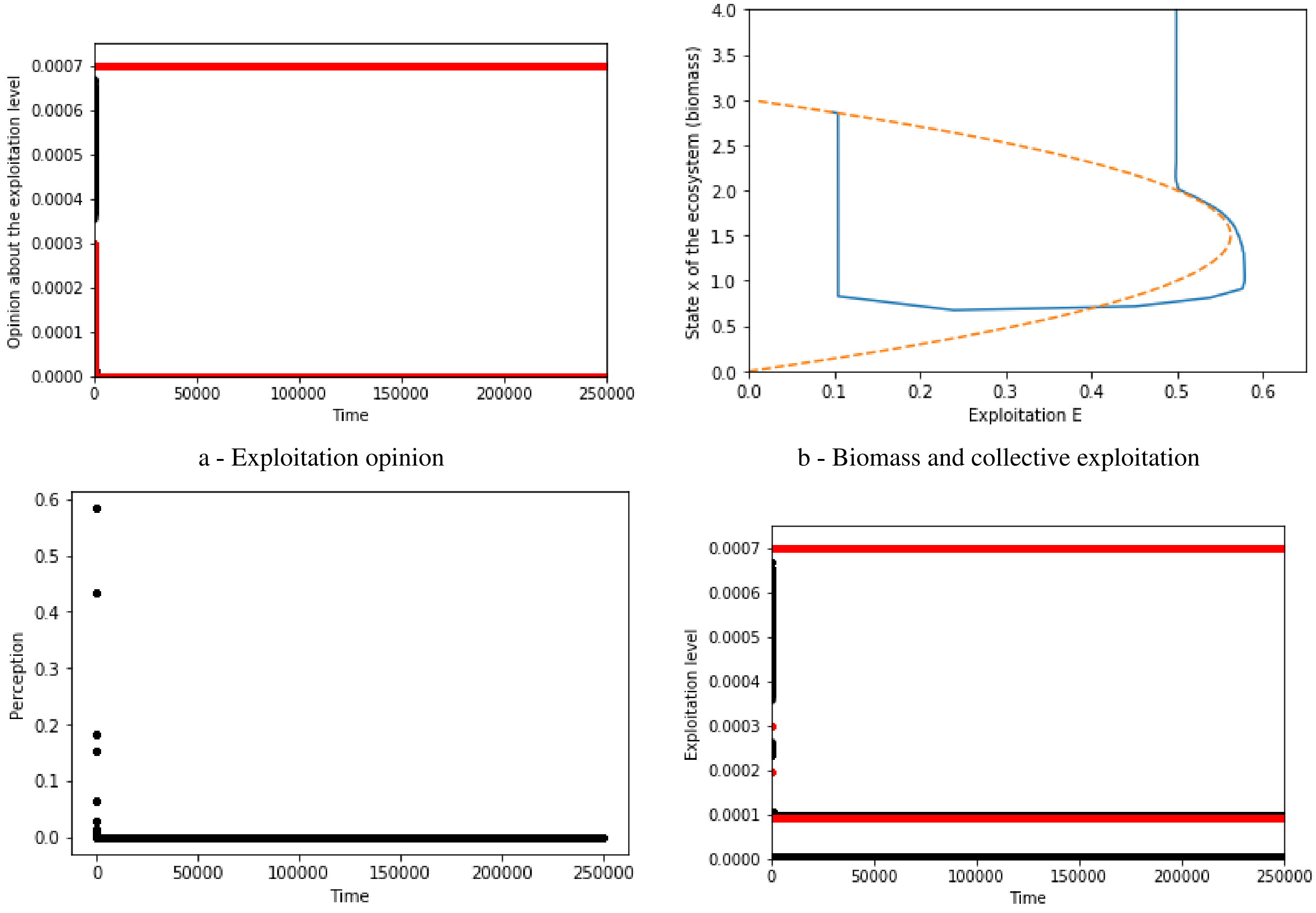

a - Exploitation opinion

b - Biomass and collective exploitation

c - Perception of moderate users

d - Individual exploitations

Figure S4: Long-term simulations, low perception case. The equilibrium obtained in Figure 5 (point E) is a stable equilibrium obtained very quickly. It is due the fact that the difference between final moderate opinion (around 0) and moderate exploitation is lower than D

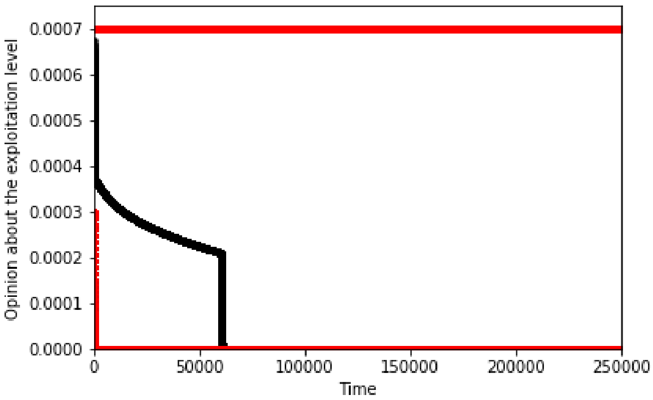

a - Exploitation opinion

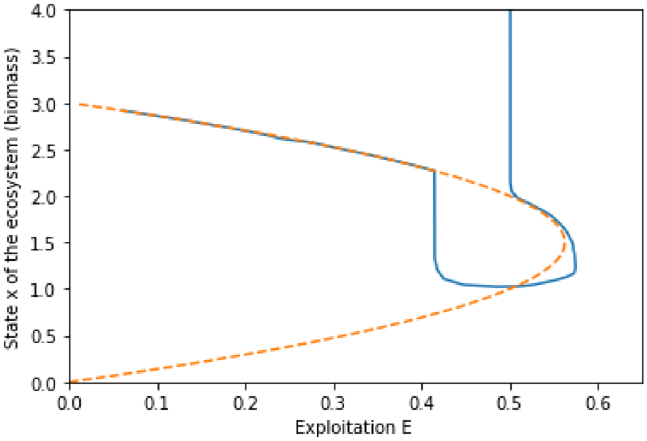

b - Biomass and collective exploitation

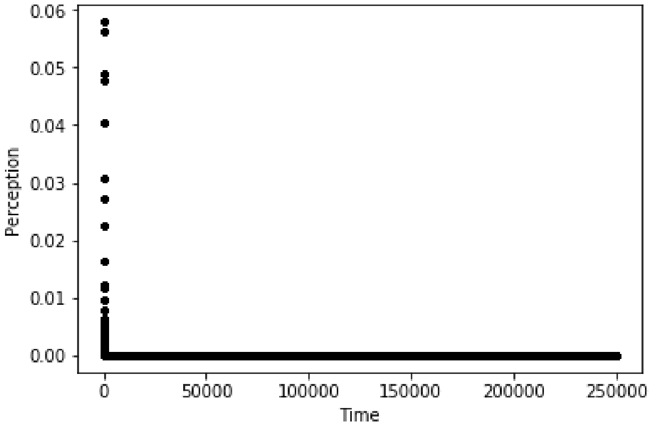

c - Perception of moderate users

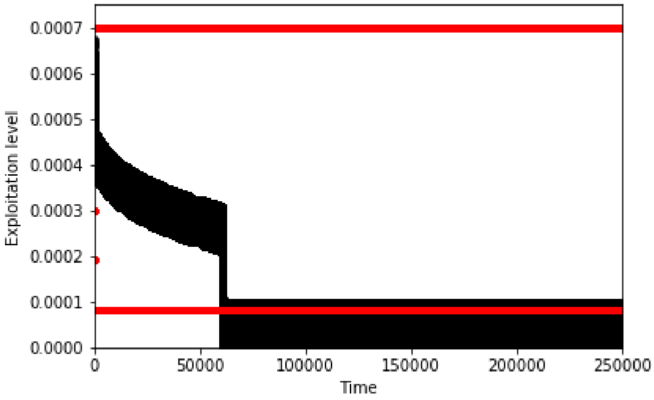

d - Individual exploitations

Figure S5: Long-term simulations, moderate perception case. The equilibrium obtained in Figure 5 (point F) is a quasi-stable equilibrium: this point is reach quickly whereas the dynamics is very slow after. Opinion of moderate users slowly evolves over time (fig a) until their opinions are quite close to the opinion of the ecological users (around time $t = 60000$). Then they are influenced by ecological users and have an opinion close to 0. Exploitation stops evolving when the difference between final moderate opinion (around 0) and moderate exploitation is lower than D

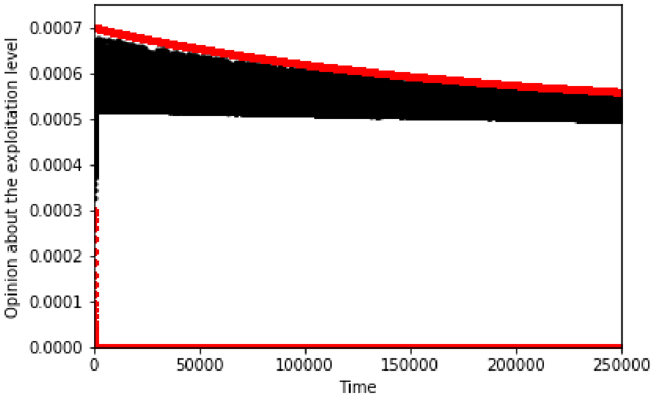

a - Exploitation opinion

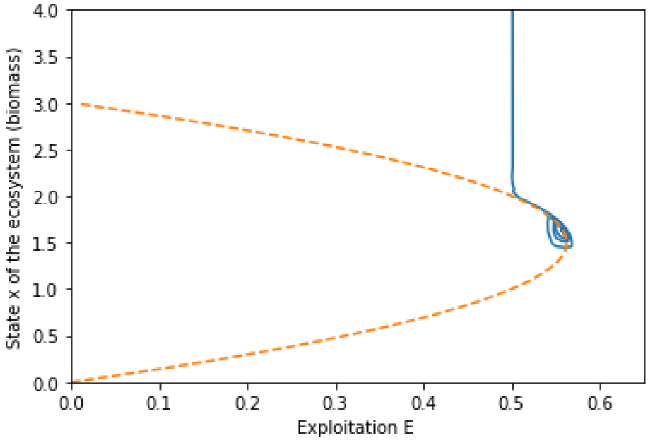

b - Biomass and collective exploitation

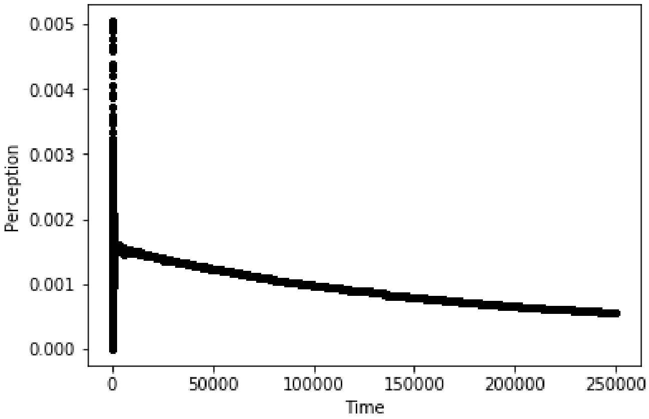

c - Perception of moderate users

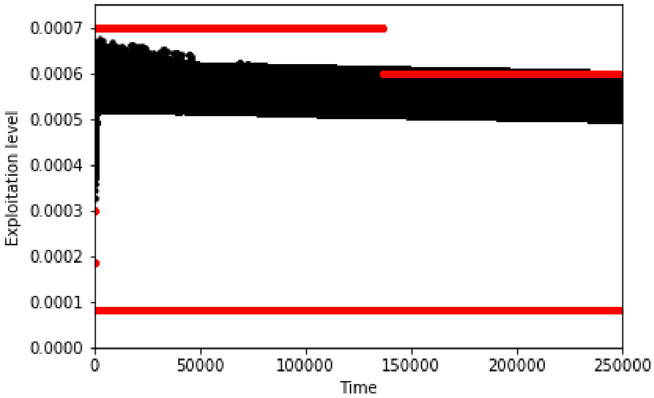

d - Individual exploitations

Figure S6: Long-term simulations, high perception case. After cyclic behavior, the equilibrium obtained in Figure 5 (point G) is a quasi-stable equilibrium: this point is reach quickly whereas the dynamics is very slow after. Opinions of moderate users and productive slowly evolve over time (fig a) until their opinions are quite close. Productive users are more influenced because of the high perception. However, the dynamics is very long (not represented here) but the convergence process is similar as the low ad moderate perception cases: exploitation stops evolving when the difference between final moderate opinion (around 0) and moderate exploitation is lower than D

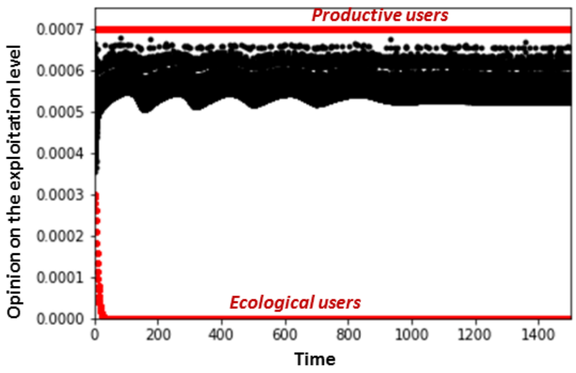

a - Exploitation opinion

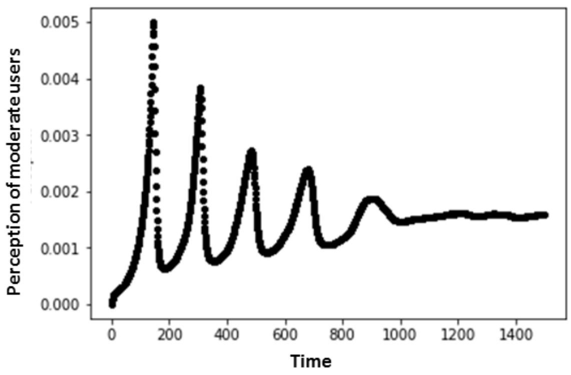

b - Perception of the ecological state

Figure S7: Opinion (fig. a) and perception (fig. b) dynamics in the case of high perception (see also the green curve in Figure 5). Opinion of moderate users (black points) oscillated between opinion of productive users (red points) due to their influence on moderate users and the right level of exploitation considering the perceived alarming state of the ecological system.

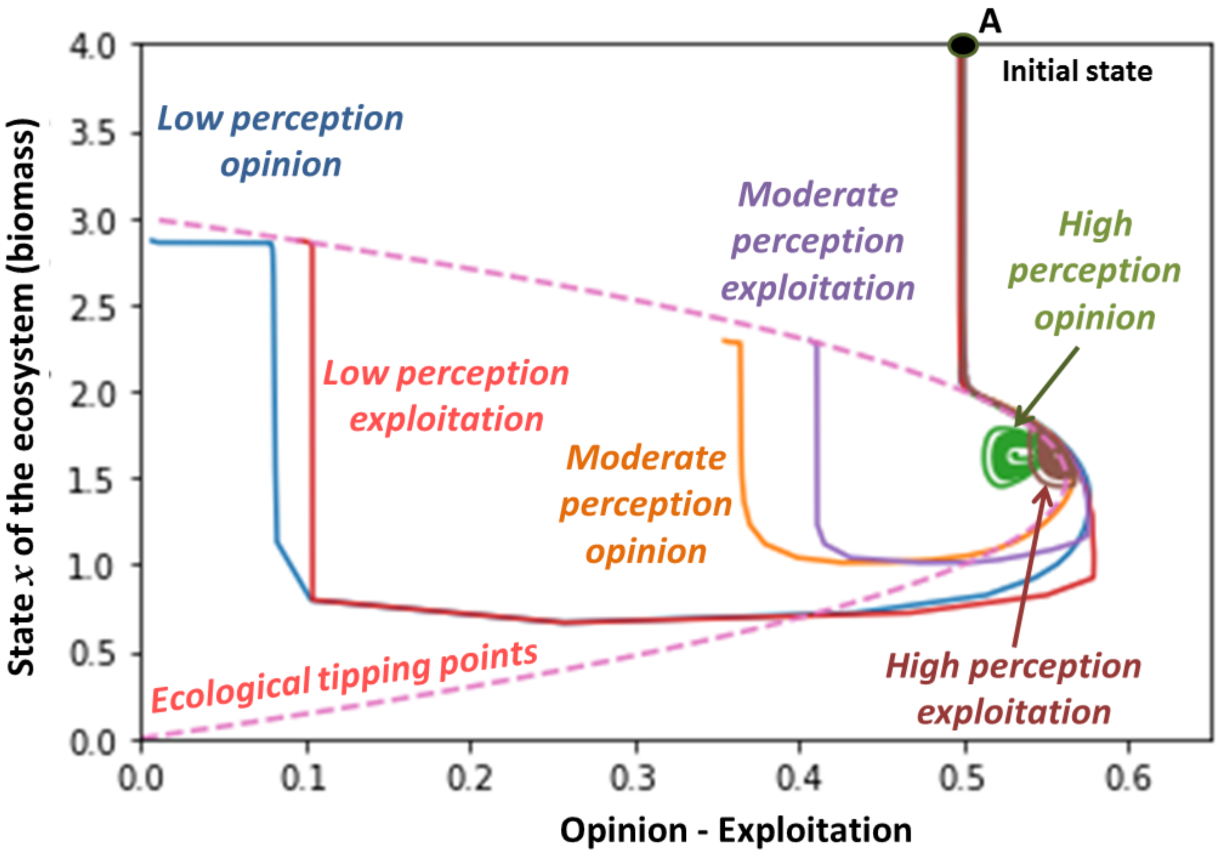

Figure S8: Dynamics of opinion and exploitation for different perception. The opinion dynamics represented here corresponds to the sum of the opinions of all agents. At a glance, the dynamics of opinions is shifted (on the left) according to the dynamics of exploitation because of the cognitive dissonance that allows difference between opinion and exploitation.

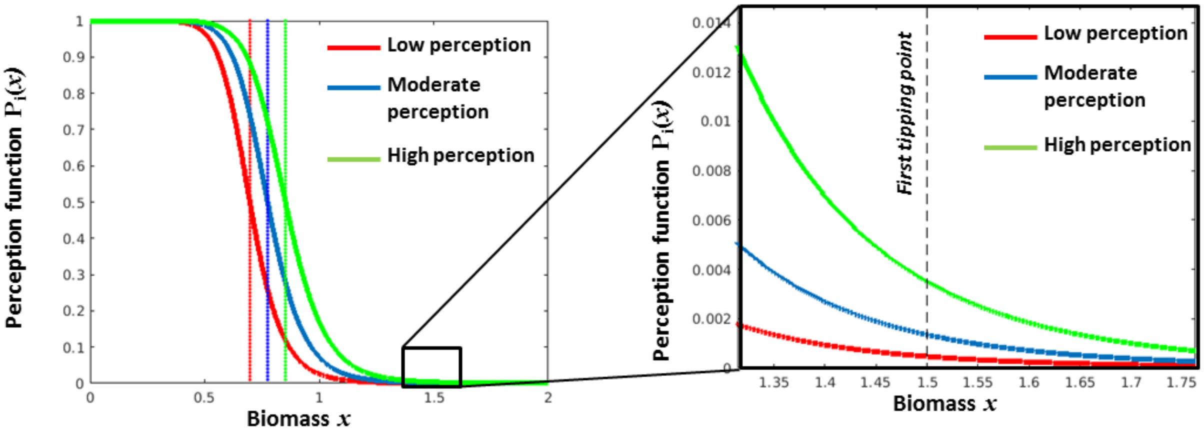

Figure S9: Perception function for different value of θ. The highest the value of θ, the highest the value of the perception function for a given value of the biomass. On the left part, a zoom of the perception function is plotted around $x = 1.5$ that corresponds to the tipping point B on Figure 5. At this point, the "‘high perception"’ value is 10 times higher than the "‘low perception"’ value.
