# Supplementary material for: Exploring non-linear transition pathways in social-ecological systems
Source: Sci Rep. 2020 Mar 5;10:4136. doi: 10.1038/s41598-020-59713-w (PMC7058029; doi:10.1038/s41598-020-59713-w)
Supplement: Supplementary file 2 — Supplementary Information2. [file 41598_2020_59713_MOESM2_ESM.pdf]

# Beyond tipping points: exploring non-linear transition pathways of social-ecological systems: Supplementary Information: case of similar perception

**Jean-Denis Mathias[1,*], John M. Anderies[2,3,4,+], Jacopo Baggio[5,6,+], Jennifer Hodbod[7,+], Sylvie Huet[1,+], Marco A. Janssen[2,4,+], Manjana Milkoreit[8,+], and Michael Schoon[2,4,+]**

[1]Université Clermont Auvergne, Irstea, UR LISC, Centre de Clermont-Ferrand, F-63178 Aubière, France
[2]School of Sustainability, Arizona State University, Wrigley Hall, 800 Cady Mall 108, Tempe, AZ 85281, United States of America
[3]School of Human Evolution and Social Change, Arizona State University, Tempe, AZ 85281, United States
[4]Center for Behavior, Institutions and the Environment, Arizona State University, Tempe, AZ 85281, United States
[5]School of Politics, Security and International Affairs, University of Central Florida, Orlando, 32816, United States
[6]Sustainable Coastal System Cluster, National Center for Integrated Coastal Research, University of Central Florida, Orlando, 32816, United States
[7]Department of Community Sustainability, Michigan State University, 480 Wilson Road Room 310 B, East Lansing, MI 48824, United States of America
[8]Department of Political Science, Purdue University, 100 N University Street, West Lafayette, IN 47906, United States of America
[*]jean-denis.mathias@irstea.fr
[+]these authors contributed equally to this work

## ABSTRACT

This Supplementary Information presents the case of the similar perception.

We consider that all users have the same perception of the ecological state and that opinions of users change according to 1) these ecosystem state perceptions and 2) their social interactions. Due to this perception process, despite that users' initial opinions are ranged between the opinions of ecological and productive users, their opinions may evolve towards an exploitation opinion lower than initial ecological users until the limit opinion of no exploitation. In this case, social tipping points are either due to social interactions (as it was the case in the previous section); or the perception of alarming ecological states. In the case of very low perception, results are quite similar to the case described in the previous section (not represented here for the sake of simplicity). On the other hand, a very high perception leads the population to behave like ecological users. We present the case where all agents have the same moderate perception on Figure S10. In both cases, there is a sudden social change of the state (around $time = 120 - 150$) due to instability of the system (left figures): opinions of the moderate users (in black) are fluctuating until a tipping towards engaged users (in red) depending on the tipping case: ecological users for case 1 (fig a) when ecological users have influenced all the moderate users and defined a strongly ecological norm, and productive users for case 2 (fig b) when productive users have influenced all the moderate users and defined a strongly productive behavior. However, unlike previous case, perception of the ecological state significantly drives the second part of the dynamics. In the first case (fig a), the perception is very low, yielding a similar result of the previous case (fig 3a) whereas all agents perceive an alarming ecological state in the second case (fig b) due to a high exploitation of the ecosystem. It leads to a new state where opinions keep fluctuating at a lower exploitation. Note that, if we continue simulations, there are two possible convergences (at infinite time) because of the perception process: either there is a long transient convergence (with a convergence of $e^{-10^{-6}t}$, $t$ being the time) or a rapid convergence towards a non-nil exploitation because of the cognitive dissonance that allows a non-nil exploitation (see Fig S4, S5 and S6 in SI for more explanations).

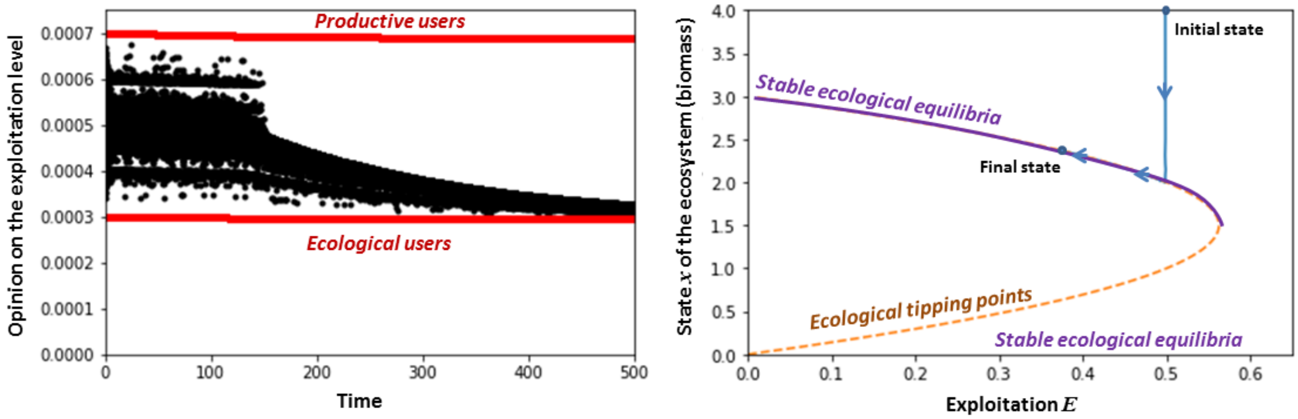


a - Transition pathway 1

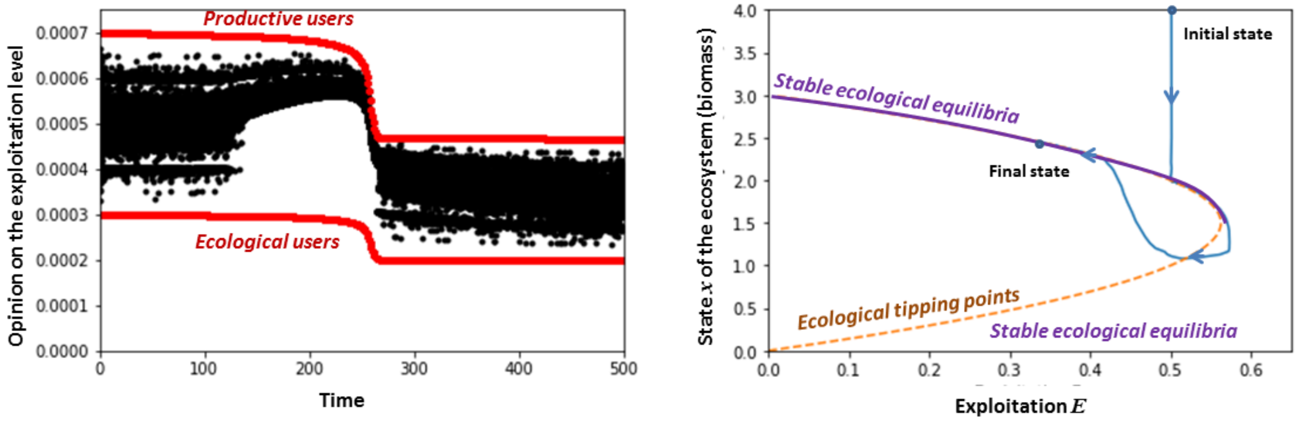


b - Transition pathway 2

**Figure S10: Transition pathways with the same perception of all agents that yield two final ecological states.** In both cases, there is a sudden social change of the state (around $time = 120 - 150$) due to instability of the system (left figures). However, unlike previous case, perception of the ecological state significantly drives the second part of the dynamics. In the first case (fig a), the perception is very low, yielding a similar result of the previous case (fig 3a) whereas all agents perceive an alarming ecological state in the second case (fig b) due to a high exploitation of the ecosystem. It leads to a new state state where opinions keep fluctuating at a lower exploitation.
